# Modularly engineering *Rhodotorula toruloides* for α-terpineol production

**DOI:** 10.3389/fbioe.2023.1310069

**Published:** 2024-01-19

**Authors:** Liting Lyu, Qiongqiong Chen, Haizhao Xue, Sumayya Mustafa, Aabid Manzoor Shah, Qitian Huang, Yue Zhang, Shuang Wang, Zongbao Kent Zhao

**Affiliations:** ^1^ Laboratory of Biotechnology, Dalian Institute of Chemical Physics, Dalian, China; ^2^ MOE Key Laboratory of Bio-Intelligent Manufacturing, School of Bioengineering, Dalian University of Technology, Dalian, China; ^3^ University of Chinese Academy of Sciences, Beijing, China

**Keywords:** *Rhodotorula toruloides*, modularly engineering, CRISPR/Cas9, monoterpenoid, α-terpineol

## Abstract

α-Terpineol is a monoterpenoid alcohol that has been widely used in the flavor, fragrance, and pharmaceutical industries because of its sensory and biological properties. However, few studies have focused on the microbial production of α-terpineol. The oleaginous yeast *Rhodotorula toruloides* is endowed with a natural mevalonate pathway and is a promising host in synthetic biology and biorefinery. The primary objective of this work was to engineer *R. toruloides* for the direct biosynthesis of α-terpineol. The improvement in monoterpenoid production was achieved through the implementation of modular engineering strategies, which included the enhancement of precursor supply, blocking of downstream pathways, and disruption of competing pathways. The results of these three methods showed varying degrees of favorable outcomes in enhancing α-terpineol production. The engineered strain 5L6HE5, with competitive pathway disruption and increased substrate supply, reached the highest product titer of 1.5 mg/L, indicating that reducing lipid accumulation is an efficient method in *R. toruloides* engineering for terpenoid synthesis. This study reveals the potential of *R. toruloides* as a host platform for the synthesis of α-terpineol as well as other monoterpenoid compounds.

## 1 Introduction

Terpenoids, the most diverse class of organic compounds, contain more than 70,000 distinct molecules with a substantially broader range of biological functions. The two most used precursors for nearly all terpenoids are dimethylallyl diphosphate (DMAPP) and isopentenyl diphosphate (IPP), which are synthesized either via the mevalonate (MVA) pathway or the 1-deoxy-D-xylulose 5-phosphate (DXP) pathway (Christianson, 2007; Daletos et al., 2020; Liu et al., 2021b). Plant-driven terpenoids encounter sustainability challenges due to their low yield and extremely high cost of the final purified product. The chemical synthesis of terpenoids is challenging due to their complicated structure. The rapid development in metabolic engineering techniques together with the latest synthetic biological tools to be applied to microbial hosts provides an alternative approach to the aforementioned sustainability issues regarding cost-effectiveness and yield production (Ren et al., 2020).

Terpineols (C_10_H_18_O) are monocyclic monoterpenoid tertiary alcohols. α-Terpineol is an isomer that widely exists in natural sources. α-Terpineol, like other monoterpenoids, has applications in the flavor and fragrance industries. In the last several years, data have been published linking this chemical to a wide range of biological characteristics, including anti-microbial, antioxidant, anti-inflammatory, anti-carcinogenic, and anticonvulsant effects (Khaleel et al., 2018; Sales et al., 2020). Except for direct extraction of terpenoids from plants or isolation from essential oils, chemical synthesis and biocatalytic production of α-terpineol have also been proposed previously. The conventional chemical method commonly employed for the synthesis of α-terpineol typically entails the hydration of α-pinene or crude turpentine oil using aqueous mineral acid (Sales et al., 2020). The majority of α-terpineol biocatalysts involve the biotransformation of their monoterpene counterparts like limonene and alpha- and beta-pinenes (Vespermann et al., 2017; Sales et al., 2018). However, this approach often results in the formation of undesired chemical byproducts.

*R. toruloides* is an oleaginous yeast that possesses its own mevalonate (MVA) pathway. *R. toruloides* has a powerful lipid metabolic machinery to drive the carbon flux for lipid production, as evidenced by the fact that cellular contents of carotenoids and lipids can reach 0.1% and 70%, respectively, on a dry cell mass basis (Ratledge and Wynn, 2002; Li et al., 2007). Due to the excellent consumption of lignocellulosic feedstock, *R. toruloides* has become a promising host in biorefinery and synthetic biology (Wen et al., 2020; Zhao et al., 2022). In recent years, this yeast has been engineered to produce terpenoids including monoterpene (limonene, etc.), sesquiterpene (bisabolene, etc.), and diterpene (*ent*-kaurene, etc.) at a titer ranging from mg/L to g/L (Geiselman et al., 2020; Liu et al., 2021a; Walls et al., 2023). Conventional strategies are often used in *R. toruloides* engineering, like overexpression of rate-limiting enzymes and increase in the copy number of terpenoid synthase genes (Geiselman et al., 2020; Liu et al., 2021a). In our previous investigation, a reduction of lipid accumulation was observed with the inactivation of lipid droplet structural proteins in *R*. *toruloides*, while the production of carotenoids was improved (Jiao et al., 2021). Particularly, the knockout of the perilipin gene *LDP1* led to a 13% decrease in lipid content and a 93% increase in carotenoid content. This study aims to explore the potential of *R. toruloides* to directly produce monoterpenoid α-terpineol through modular engineering, including the enhancement of precursor supply, the blocking of downstream pathways, and the disruption of competing pathways.

## 2 Materials and methods

### 2.1 Strains and media

All strains used in this study are listed in Supplementary Table S1. *R. toruloides* NP11, a haploid yeast strain of CGMCC 2.1389, was isolated in our laboratory. Yeast cells were cultured in the yeast extract–peptone–dextrose (YPD) medium (glucose 20 g/L, yeast extract 10 g/L, and peptone 20 g/L) at 30 C. *Escherichia coli* DH10B and *Agrobacterium tumefaciens* AGL1 used for routine molecular manipulation were cultured in the Luria–Bertani medium (NaCl 10 g/L, tryptone 10 g/L, and yeast extract 5 g/L) at 37°C and 30°C, respectively. Antibiotics were used at the following concentrations: kanamycin 50 μg/mL, hygromycin 50 μg/mL, nourseothricin 50 μg/mL, zeocin 50 μg/mL, and cefotaxime 300 μg/mL. For yeast culturing, the engineered strains were cultured in Y_10_T_20_D_50_ medium (tryptone 20 g/L, yeast extract 10 g/L, and glucose 50 g/L, pH 6.0) or nitrogen-limited (NL) medium (glucose 50.0 g/L, yeast extract 1.1 g/L, (NH_4_)_2_SO_4_ 0.1 g/L, MgSO_4_ 1.5 g/L, and KH_2_SO_4_ 1.0 g/L at a C/N ratio of 125, pH 6.0).

Yeast extract, peptone, and tryptone were acquired from Oxoid (Basingstoke, Hampshire, UK), while other chemicals were acquired from Bonuo Biological and Chemical Reagent Co. Ltd. (Dalian, China).

### 2.2 Plasmid construction

The rational sgRNAs, corresponding to particular loci of target genes, were designed according to the previous protocol to obtain the gene-targeted strain (Jiao et al., 2019). The *α-terpineol synthase* (*aTS*) gene from *Vitis vinifera* (GenBank ID: AAS79352.1) was codon-optimized and synthesized by Synbio Technologies (Suzhou, China). This synthetic gene has been submitted to NCBI (Accession Number: OQ983656). The *truncated 3-hydroxy-3-methylglutaryl-CoA reductase* (*tHMG1*) gene was amplified from the plasmid p424-tHMG1 (Zhou et al., 2012). *ERG20* was amplified from the *Saccharomyces cerevisiae* genome and then mutated to *ERG20* ^*ww*^ (F96W/N127W). All plasmids were constructed using the restriction-free cloning method (Unger et al., 2010). The plasmid extraction kit and DNA gel purification kit were purchased from Sangon Biotech Co., Ltd. (Shanghai, China), and polymerase chain reaction (PCR) enzymes were purchased from Takara Bio (Dalian, China). All the sequences of plasmids and primers are provided in Supplementary Tables S2, 3.

### 2.3 Transformation and screening

The correct plasmids were transformed into *Agrobacterium tumefaciens* AGL1 by electroporation, and strains were selected on LB plates supplemented with 50 μg/mL kanamycin. Transformation of *R. toruloides* was done according to a published *Agrobacterium tumefaciens*-mediated transformation (ATMT) method (Lin et al., 2014). PCR analysis of the *R. toruloides* colony for transformant verification was assayed according to previous reports (Lin et al., 2012; Lin et al., 2014). The primer pairs used in this study are shown in Supplementary Table S3.

### 2.4 Genome walking

The genome walking protocol was followed as previously reported (Liu and Chen, 2007). High-quality genome DNA was extracted from the engineered red yeast based on the standard protocol (Lin et al., 2014). Amplified PCR products were purified and sequenced following second thermal asymmetric interlaced PCR. DNA sequences were blasted against the *R. toruloides* NP11 local genomic database to determine the accurate insertion sites.

### 2.5 Well plate and shake-flask culturing

Seed cultures of 5 mL of YPD medium were prepared by inoculating a single colony and incubating it for 24 h. A measure of 200 μL of the seed culture was inoculated into each column of a 24-well culturing plate. Subsequently, 1,800 μL of Y_10_T_20_D_50_ media was added, and the plate was incubated at 30°C for 144 h with agitation at a speed of 200 rpm.

For shake-flask culturing, seed cultures were prepared by inoculating a single colony from selected culture plates into 5 mL YPD medium and incubating it for 24 h followed by reviving using 1 mL volume into 50 mL YPD. Then, 5 mL of these seed cultures was transferred into 45 mL Y_10_T_20_D_50_ medium in a 250-mL shake flask kept at 30°C for 144 h at 200 rpm. Fermentation was performed in triplicate.

### 2.6 Extraction and quantification of α-terpineol

A measure of 2 mL of the fermented liquor was taken, and the same amount of n-hexane was added. The organic solvent and medium mixture were incubated at 200 rpm and 25°C for 1 h before being centrifuged at 4,000 g for 5 min. Following the extraction process, a measure of 500 μL from the n-hexane layer was carefully transferred into a vial made of darkened glass in order to facilitate subsequent analysis. α-Terpineol was identified by GC-MS (Agilent Technologies 7890A GC system equipped with a 5975C insert 143 XL EI/CI MSD detector) with a DB-WAX column (30 m × 0.32 mm × 0.25 μm). The amount of α-terpineol was determined from linear calibration curves (Supplementary Figure S1). The standard α-terpineol was purchased from Solarbio (China, GC ≥ 99.0%). For GC analysis, 1 μL of the *n*-hexane sample was injected with a split ratio of 10:1, and nitrogen was used as the carrier gas with a flow rate of 1 mL/min. The injector temperature and detector temperature were maintained at 250°C and 260°C, respectively. The oven temperature was maintained as follows: 80°C for 1 min and sequentially increased at the rate of 10°C/min to 180°C and 10°C/min to 250°C.

### 2.7 Copy number quantification

High-quality genomic DNA of engineered *R. toruloides* was extracted following the previous protocol (Lin et al., 2014) and then quantified using NanoDrop™ 1000 (Thermo Fisher Scientific). To determine the relative copy number of the introduced transgenes, quantitative PCR was performed using the ChamQ Universal SYBR qPCR Master Mix (Vazyme Biotech Co., Ltd., China) on a real-time quantitative PCR system (Roche, Switzerland) using the manufacturer’s instructions. Each reaction required 20 μL and was set up in triplicate with 100 ng of genomic DNA as a template. PCR products (approximately 3 kb) spanning the qPCR amplicons were amplified from genomic DNA for the native sequences and plasmid DNA for the transgenes. These PCR products were purified using the FastPure Gel DNA Extraction Kit (Vazyme Biotech Co., Ltd., China) and used to generate standard curves for each qPCR primer set (Supplementary Figure S2). Since *tHMG1*, *ERG20* ^*ww*^, and *HYG* were designed into one cassette for co-expression, the transgene *HYG* was used to represent the first two. The standard curves were used to calculate the copy number of transgenes relative to the native gene *ACTIN* (GenBank ID: RHTO_03560) and the transgene *HYG*. The primers used are listed in Supplementary Table S2.

## 3 Results

### 3.1 Re-casting the α-terpineol biosynthesis by *R. toruloides* into three modules

**FIGURE 1:**
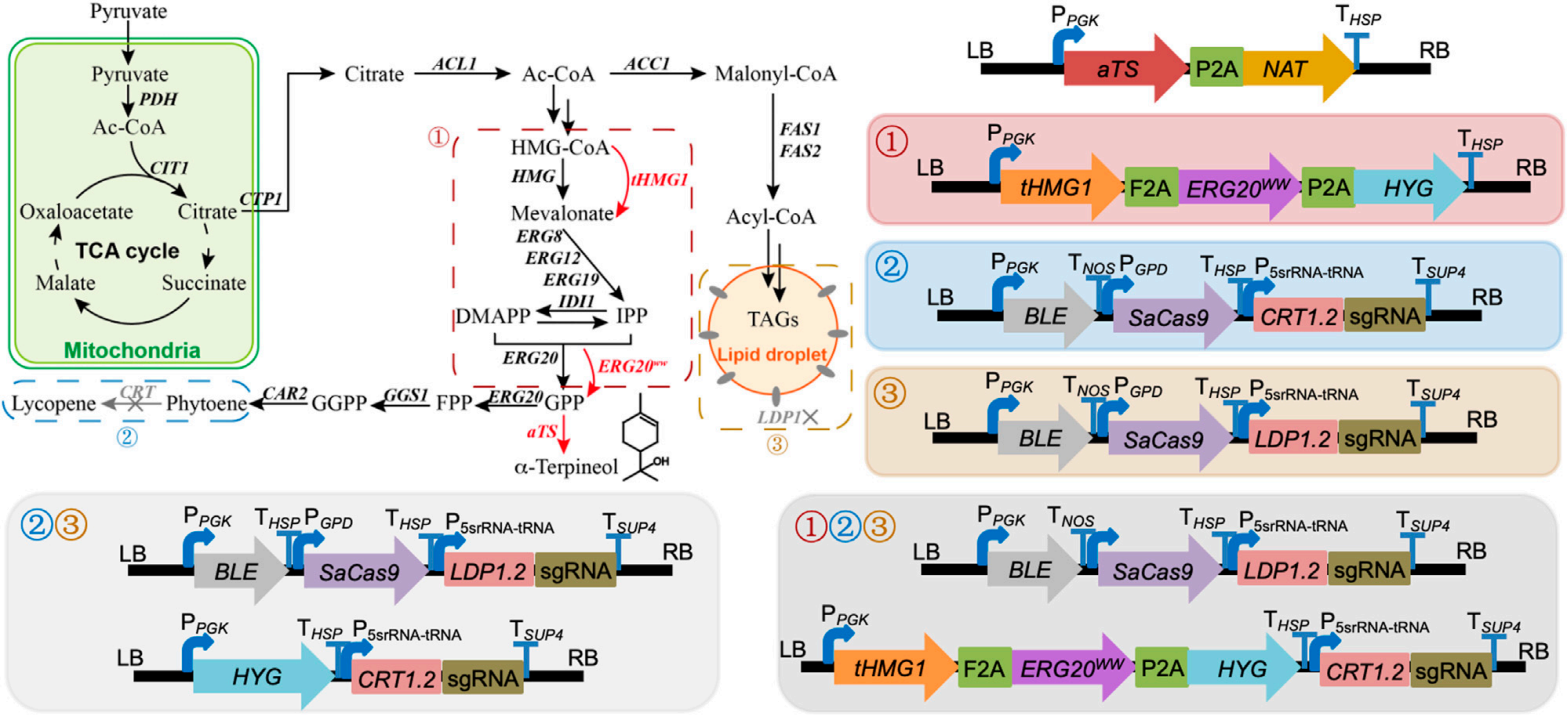
Modular engineering of *Rhodotorula toruloides* for α-terpineol production. ①, ②, and ③ represent the modules of precursor supply enhancement, downstream pathway blocking, and competing pathway disruption, respectively. Combined modules ①② and ①③ were assembled by directly expressed cassettes under each module in *R. toruloides* that is not shown. *PDH*, pyruvate dehydrogenase; *CTP1*, citrate transporter; *ACL1*, ATP citrate synthase; *ACC1*, acetyl-CoA carboxylase; *HMG1*, *3-hydroxy-3-methylglutaryl-CoA reductase*; *tHMG1*, *truncated HMG1*; *IDI1*, *isoprene diphosphate isomerase*; *ERG8, phosphomevalonate kinase*; *ERG12*, *mevalonate kinase*; *ERG19*, *mevalonate pyrophosphate decarboxylase*; *ERG20*, *bifunctional FPP synthase*; *ERG20* ^*ww*^, *ERG20* mutant (F96W/N127W); *GGS1*, g*eranylgeranyl diphosphate synthase*; *CAR2*, bifunctional lycopene cyclase/phytoene synthase; *CRT*, phytoene dehydrogenase; *aTS*, α-terpineol synthase; *FAS1* and *FAS2*, fatty acid synthase 1 and 2; IPP, 3-isopentenyl pyrophosphate; DMAPP, dimethylallyl diphosphate; GPP, geranyl pyrophosphate; FPP, farnesyl pyrophosphate; GGPP, geranylgeranyl pyrophosphate; TAGs, triacylglycerols; TCA, tricarboxylic acid.

To obtain an engineered strain of *R. toruloides* with the ability to synthesize α-terpineol, *aTS* was initially incorporated into the *R. toruloides* genome via the ATMT protocol. Using this modified strain as a basis, three modular pathways (the enhancement of precursor supply, the blocking of downstream pathways, and the disruption of competing pathways) were adjusted to optimize the production of *α-terpineol synthase* (Figure 1). Particularly, in the module of precursor supply enhancement, *tHMG1* and *ERG20* ^*ww*^ were co-expressed, which proved to be helpful in yeast monoterpene production (Ignea et al., 2014). In the module of downstream pathway blocking, the CRISPR/Cas9 system was used to knock out the *CRT* gene (GenBank ID: RHTO_04602) encoding phytoene dehydrogenase. Thus, the synthesis of carotenoids was blocked, and the engineered strains showed white color. In the module of competing pathway disruption, the lipid droplet structural protein, namely, perilipin (Ldp1, GenBank ID: RHTO_05627), was inactivated by the CRISPR/Cas9 system. This strategy was based on our previous study, which suggested a reduction of lipid accumulation and higher production of carotenoids via knocking out the *LDP1* gene (Jiao et al., 2021). The sgRNAs of two targeted genes (*CRT* and *LDP1*) were constructed according to the protocol mentioned in Jiao et al. (2019).

### 3.2 α-Terpineol production and its extraction

**FIGURE 2:**
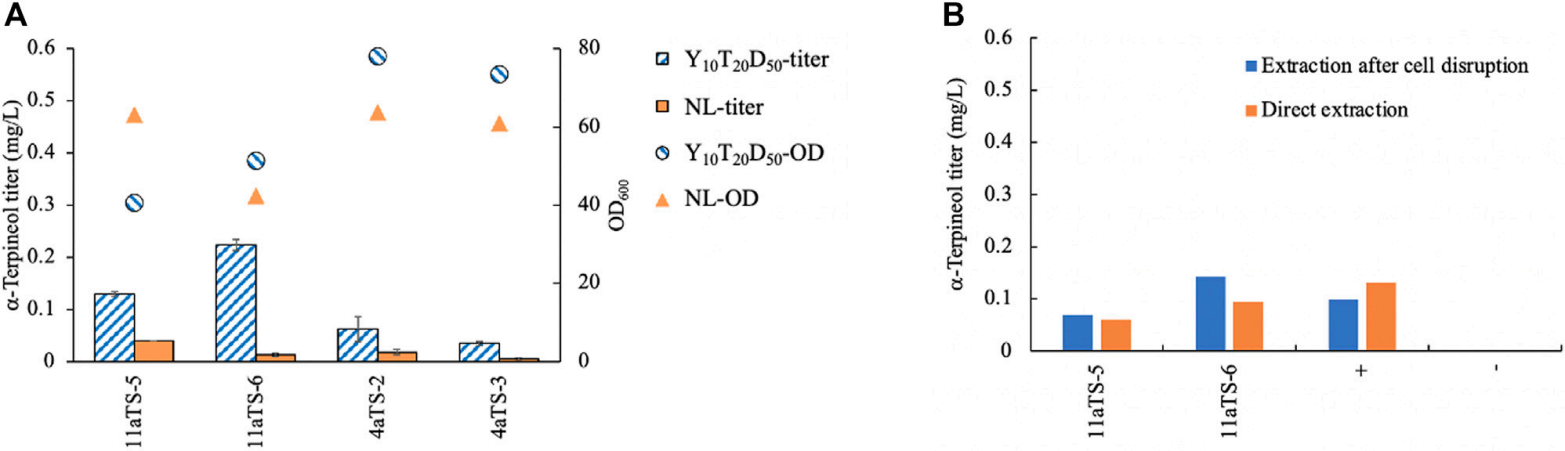
Comparison of different methods for yeast culturing **(A)** and production extracting **(B)**. 11aTS-5 and 11aTS-6, engineered strains by expressing the α-terpineol synthase gene *aTS* in haploid NP11; 4aTS-2 and 4aTS-3, engineered strains by expressing *aTS* in diploid CGMCC 2.1389; +, wild-type NP11 cultured in Y_10_T_20_D_50_ medium with the addition of 1 μM (0.15 mg/L) standard α-terpineol; wild-type NP11 cultured in Y_10_T_20_D_50_ medium.

To test which medium was suitable for α-terpineol production, two types of media, Y_10_T_20_D_50_ and NL, were chosen for culturing. YPD medium with a high concentration of glucose was often used in terpene biosynthesis in order to accumulate a higher level of biomass (Zhuang et al., 2019; Geiselman et al., 2020; Kirby et al., 2021). Here, peptone was replaced by tryptone to speed up the fermentation process. Because terpene is a secondary metabolite that has natural storage for lipid droplets, NL medium was also considered. The *aTS* gene was expressed in both strains of NP11 and CGMCC 2.1389. The α-terpineol titer in the Y_10_T_20_D_50_ culture was higher than that in the nitrogen-limited culture in all cases (Figure 2A), indicating a competitive relationship between lipid accumulation and isoprenoid synthesis. Therefore, the growth medium Y_10_T_20_D_50_ seemed better for α-terpineol production. Moreover, the diploid *R. toruloides* CGMCC 2.1389 produced less α-terpineol yield than the haploid NP11 strain.

Using *n*-dodecane as an organic solvent for *in situ* extraction of terpenoid products, a commonly used method, was employed in this study. However, the retention times of α-terpineol and *n*-dodecane totally overlapped and became difficult to separate by our GC–MS instrument. Due to the extremely high signal of the solvent, which might damage the detector and contaminate the ion source, *n*-hexane was used to extract the product after sampling. Since α-terpineol was an intracellular product, the question of whether or not it must be extracted by disrupting the cell was investigated. Wild-type NP11 cultured in Y_10_T_20_D_50_ medium with 1 μM (0.15 mg/L) standard α-terpineol was used as the control. All strains were grown for 96 h, after which samples were obtained. The findings indicated that the direct extraction of α-terpineol yielded comparable results to the extraction process conducted after cell destruction (Figure 2B). The control group also demonstrated the stability of α-terpineol under specific culturing conditions.

The engineered strain 11aTS-6 generated more α-terpineol than 11aTS-5 under the same conditions. However, after integrating an exogenous gene, the 11aTS-6 strain failed to develop in a continuous strain engineering experiment. The genome walking result showed that the *aTS* expression cassette was inserted in the exon of *serine/threonine-protein kinase* gene (RHTO_08159, Scaffold_9_589106), which played a central role in endocytosis in both humans and yeast (Smythe and Ayscough, 2003). It is speculated that the destruction of this gene might play a role in abnormal cell differentiation.

**FIGURE 3:**
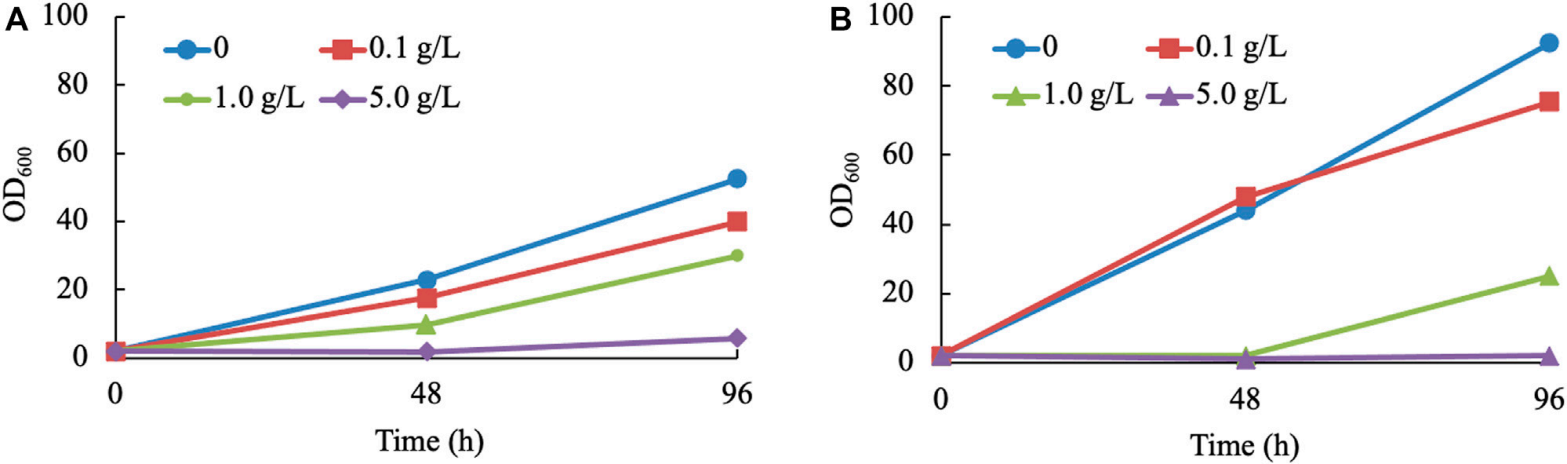
Growth of *R. toruloides* in α-terpineol-involved cultures. **(A, B)** Culturing haploid NP11 and diploid CGMCC 2.1389 under various concentrations of α-terpineol, respectively.

Considering the toxicity and instability of most monoterpenes (Alonso-Gutierrez et al., 2013), it is inevitable to detect the inhibiting effect exerted by terpineol. For economic reasons, a mixture of terpineol isomers was considered to be the inhibitor of the yeast (haploid NP11 and diploid CGMCC 2.1389) culture. Three concentration gradients of 0.1, 1.0, and 5.0 g/L were set up. The findings of the study indicate that the presence of terpineol at a concentration as low as 0.1 g/L has a discernible impact on cell development. Furthermore, it was observed that the growth of *R. toruloides* is completely suppressed when exposed to a terpineol concentration of 5.0 g/L (Figure 3). Due to the relatively low concentrations of α-terpineol in this study (under 2 mg/L), its inhibiting effect was not considered. However, when the concentration of this product is higher than 0.1 g/L, other extraction methods or manipulations to the strain need to be used. In summary, strain 11aTS-5 was chosen to perform various engineering tasks, including growing in the Y_10_T_20_D_50_ medium and subsequent direct extraction using n-hexane following the fermentation process.

### 3.3 Promoting the α-terpineol production through the modular engineering strategy

**FIGURE 4:**
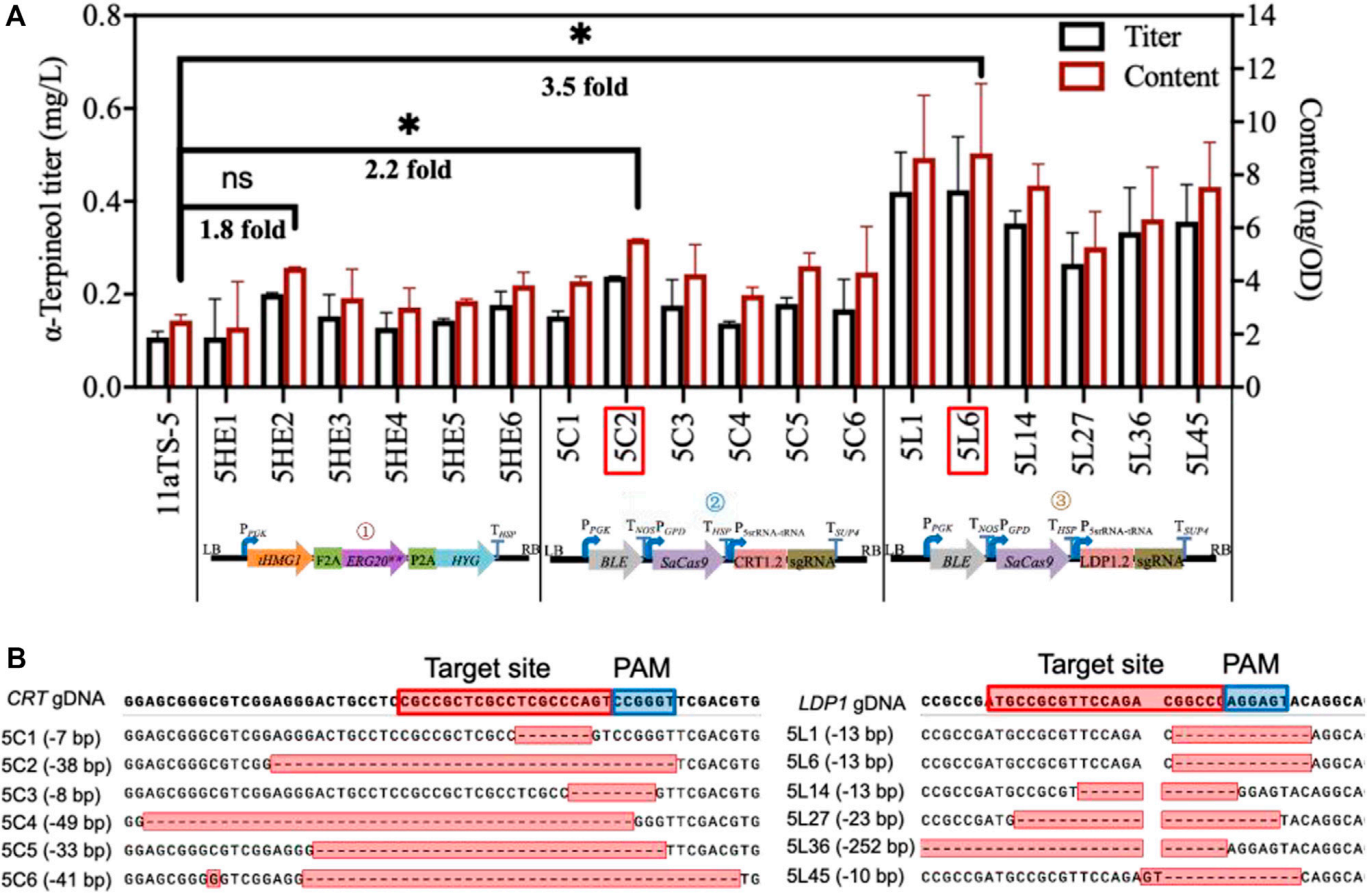
Production of α-terpineol under three modular engineering strategies. **(A)** Titer and the content of α-terpineol; **(B)** genotype of engineered strains with mutated *CRT* or *LDP1*. Significantly different values were calculated by the independent-samples t-test. * and ns represent *p* < 0.05 and no significant difference, respectively. Experiments were performed in triplicate.

The strategy of promoting α-terpineol production was organized into three modules, including the enhancement of precursor supply, the blocking of downstream pathways, and the disruption of competing pathways (Figure 1). In each module, six transformants were selected for titer and content detection of α-terpineol (Figure 4A). Since the CRISPR/Cas9 system was introduced into modules ② and ③, transformants with different mutations of the target gene were chosen (Figure 4B). Consistent with the assumption, after the engineering of the baseline strain 11aTS-5, the production of α-terpineol was improved to varying degrees (Figure 4A). Particularly, compared with the 11aTS-5 strain, the optimal strain in each module, namely, 5HE2, 5C2, and 5L6, achieved a 0.8-, 1.2-, and 2.5-fold increase in α-terpineol content, respectively.

These results signify that sufficient precursor supply and substantial and continuous carbon source increment into the α-terpineol synthesis pathway become imperative. It also shows that the reduction of lipid accumulation is an efficient strategy in monoterpene α-terpineol synthesis.

### 3.4 Further improvement in the α-terpineol production using combined strategies

**FIGURE 5:**
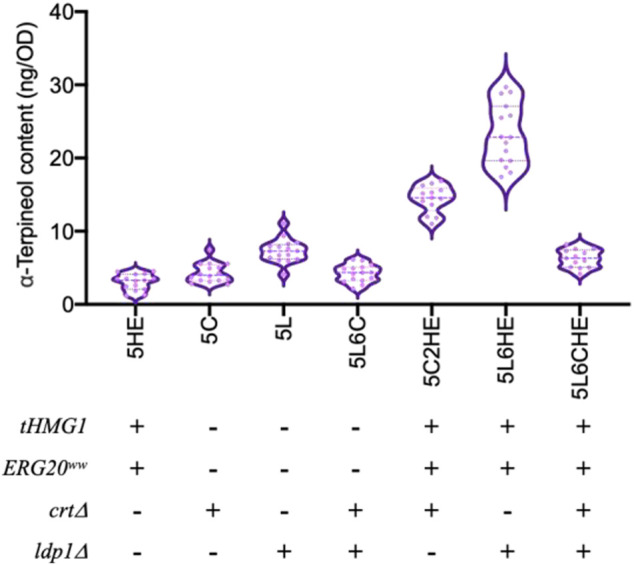
Comparison of α-terpineol production under individual or combined modular engineering strategies. + and – represent performing and not performing this gene manipulation, respectively. Under each genotype, six engineered strains cultured with three parallels were counted and analyzed.

In strain 5L6, the *tHMG1*–*ERG20* ^*ww*^ co-expression cassette and the sgRNA cassette of the *CRT* gene target were integrated individually to assemble module ①③ and module ②③ (Figure 1). For module ①②③, the above two cassettes were co-expressed in strain 5L6. As shown in Figure 5, the inactivation of both Ldp1 and Crt failed to increase the α-terpineol production as expected. However, the expression of tHmg1 and Erg20^ww^ in either the *ldp1Δ* strain or *crtΔ* strain could further improve the α-terpineol content. It indicated that tHmg1 and Erg20^ww^ were also rate-limiting enzymes in *R. toruloides*, and the synthesis of α-terpineol needs enough precursor substance. Interestingly, the detected content of α-terpineol in 5L6CHE strains, which combined three strategies, was similar to that in the *LDP1*-and *CRT*-knockout strains 5L6C. It was a widely accepted hypothesis that ATMT easily caused the insertion of multiple T-DNAs into the same chromosomal site (Tzfira, 2004). In this study, the 5L6CHE strains were obtained by co-expressing the *tHMG1*–*ERG20* ^*ww*^ cassette and the sgRNA cassette of the *CRT* gene target (Figure 1). Thus, we speculated that the CRISPR editing system interfered with the mechanism of forming high copies by ATMT.

### 3.5 Supplementation of the precursor is crucial in α-terpineol production

**FIGURE 6:**
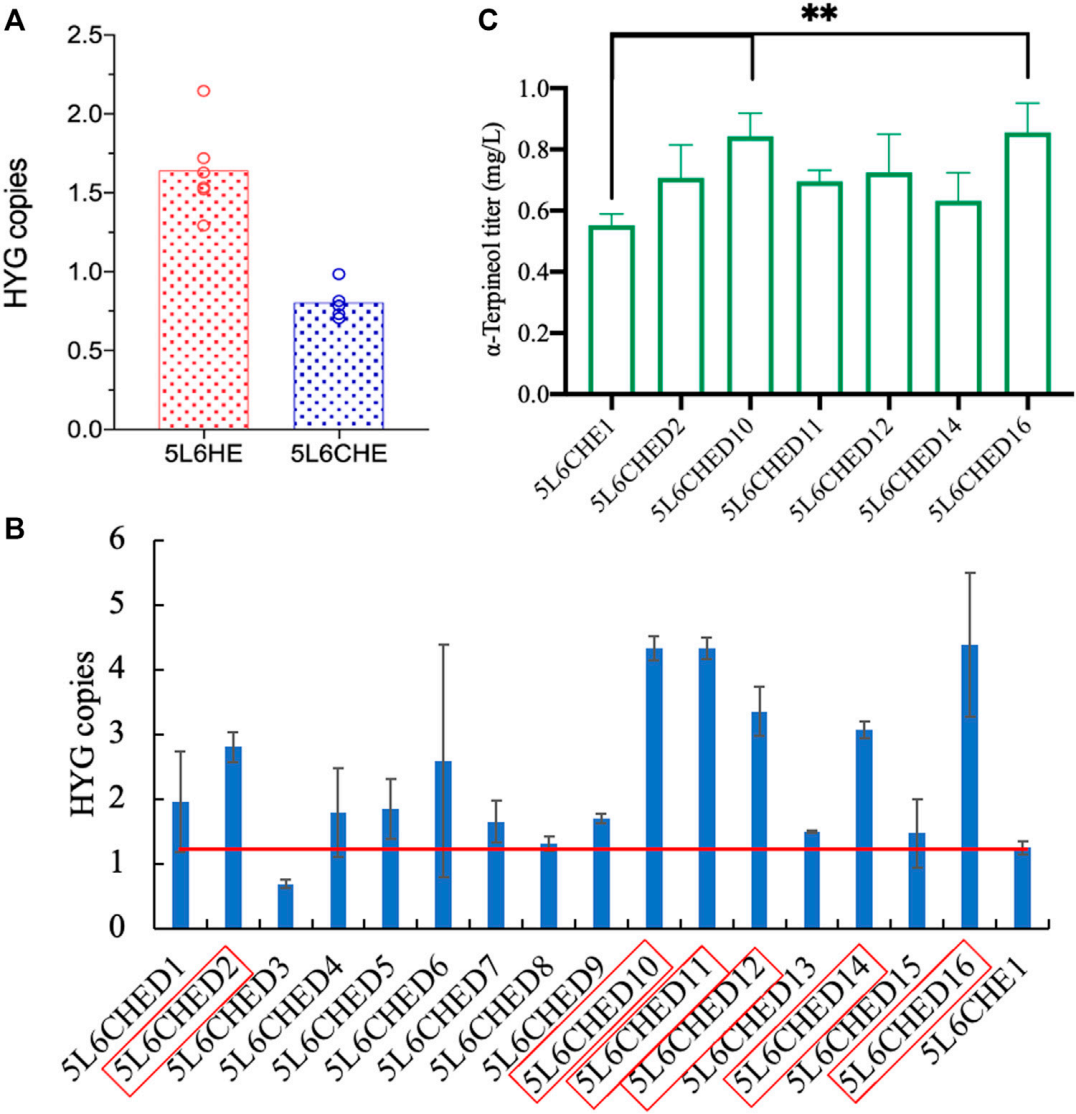
Copy number calculation and overexpression of genes *tHMG1* and *ERG20* ^*ww*^. **(A)** Comparison of *tHMG1* and *ERG20* ^*ww*^ copy numbers (represented by copy numbers of *HYG*) in 5L6HE and 5L6CHE strains; **(B)** *tHMG1* and *ERG20* ^*ww*^ copy numbers (represented by copy numbers of *HYG*) in 16 transformants with overexpression of these two genes, and the red border indicates the engineered strain chosen for product quantification; **(C)** titer of α-terpineol in six cultured strains. Significantly different values were calculated by one-way ANOVA and *post hoc* Dunnett’s test. ** represents *p* < 0.01. Experiments were performed in triplicate.

To verify the above assumption, the copy numbers of genes *tHMG1* and *ERG20* ^*ww*^ in 5L6CHE strains were first detected by qPCR. The 500-bp fragments of genes *HYG* and *ACTIN* in each strain genome were amplified to calculate the copy number of the gene *HYG*, which actually represents the copy number of genes *tHMG1* and *ERG20* ^*ww*^. Strains of 5L6HE with gene expression of *tHMG1* and *ERG20* ^*ww*^ and knockout of *LDP1* were used as the control. The results showed that the mean copy number of the *HYG* gene in 5L6CHE strains was 0.8, which was significantly lower than that in the 5L6HE strains (Figure 6A; Supplementary Table S4), which is consistent with the above assumption.

Then, the co-expression cassette of *tHMG1*–*ERG20* ^*ww*^ was incorporated into the genome of 5L6CHE strains again to overexpress tHmg1 and Erg20^ww^. After transformation, 16 transformants were selected randomly. The copy number of the *HYG* gene in each transformant was calculated. If the *HYG* copy was twice, then the starting strains 5L6CHE1, tHmg1, and Erg20^ww^ were considered to be overexpressed successfully. As a result, six strains with high copy numbers were chosen to detect the α-terpineol production (Figures 6B,C). The results revealed that the engineered strains 5L6CHED10 and 5L6CHED16 showed a significant increase in α-terpineol production, which was 1.5 times that of the starting strain 5L6CHE1.

### 3.6 Batch culturing

**FIGURE 7:**
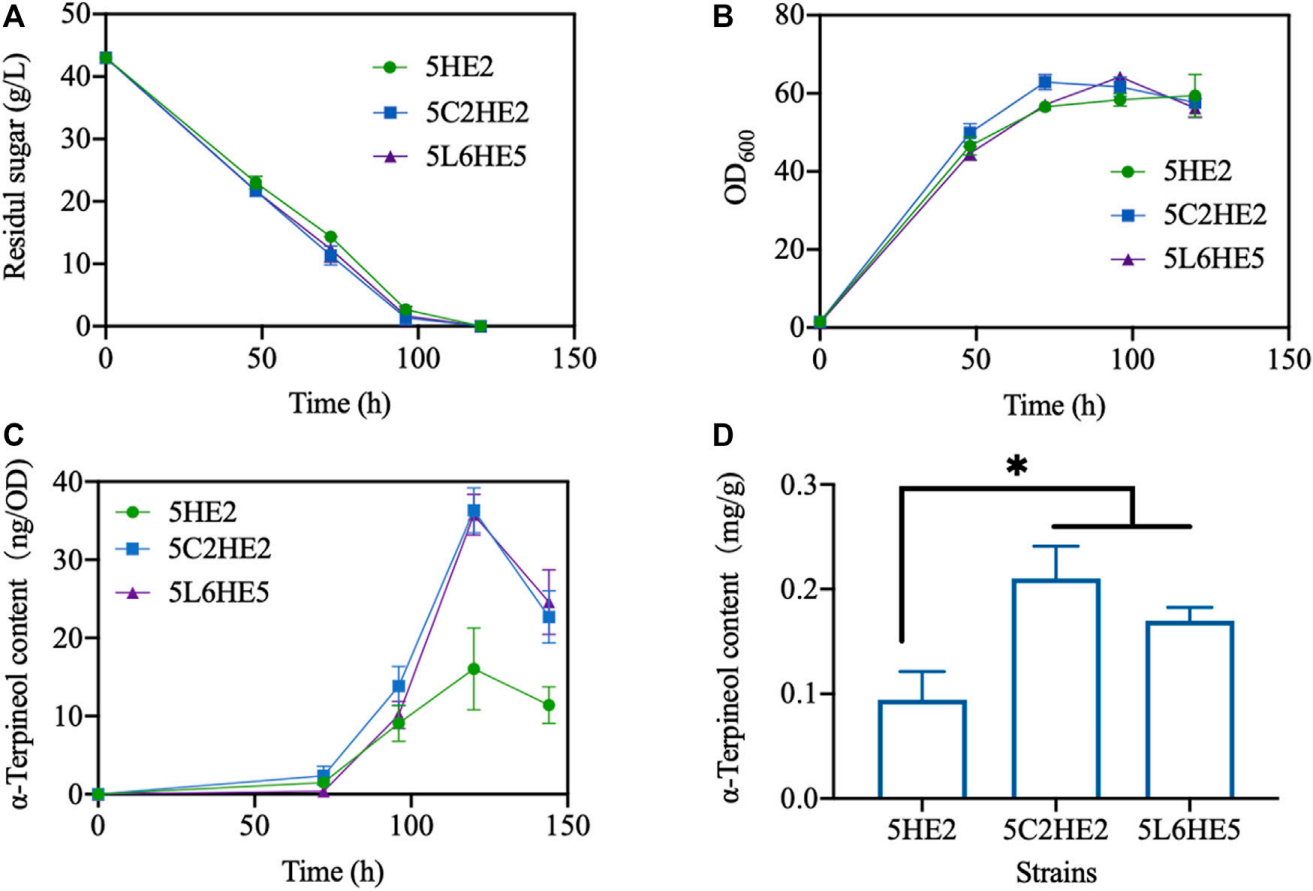
Fermentation performance of engineered strains. **(A)** Glucose consumption; **(B)** cell growth; **(C)** variation trend of α-terpineol contents during strain culturing; and **(D)** α-terpineol content at the fermentation end-point. Significantly different values were calculated by one-way ANOVA and *post hoc* Dunnett’s test. * represents *p* < 0.5. Experiments were recorded in triplicate.

Lastly, three selected engineered strains created using various strategies with the maximum content of α-terpineol were fermented using the shake-flask method (Supplementary Figure S3). To assess the efficacy of extending the culture time on enhancing product accumulation, the duration of the culture was increased from 96 h to 144 h. The experimental findings indicated that the glucose was fully metabolized within 120 h, as evidenced by a consistent OD_600_ reading (Figures 7A,B). At this stage, the content of α-terpineol was increased with time (Figure 7C). In a manner similar to the 2-mL culture method, it was observed that modified strains 5C2HE2 and 5L6HE5 exhibited a higher accumulation of the product compared to strain 5HE2. On the other hand, a decrease in α-terpineol content was observed when the culture period was increased to 144 h. Longer periods of cultivation raise the possibility that α-terpineol will evaporate or be transformed into other compounds. Inconsistent with our expectation, the production of α-terpineol in a 250-mL flask failed to be further promoted but remained the same with 2-mL culturing (data were not shown). Quantitative analysis showed that the maximum content of α-terpineol observed in 5C2HE2 and 5L6HE5 was 0.20 and 0.17 mg/g DCW, respectively (Figure 7D).

## 4 Discussion

Modular engineering strategies, including the enhancement of precursor supply, the blocking of downstream pathways, and the disruption of competing pathways, were applied to promote α-terpineol production. It was found that these three strategies improved the production of α-terpineol to a certain degree. Future applications for using engineered *R. toruloides* for α-terpineol production via expression of the *aTS* gene and successful detection of α-terpineol are envisioned in this study. Particularly, the strain 5L6HE5 with *LDP1* knock out and *tHMG1* and *ERG20* ^*ww*^ expression achieved the highest product titer, suggesting that the reduction of lipid accumulation is an efficient strategy in α-terpineol synthesis. Additionally, the synthesis and accumulation pathway of lipids was further downregulated to derive more carbon flux into the MVA pathway.

Based on these engineering strategies, the titer of α-terpineol failed to improve continuously through either supporting enough precursors or scaling up the culture (Figures 6, 7). There may be other rate-limiting enzymes in the monoterpenoid production pathway that are preventing it from functioning at full capacity. The catalysis mediated by *aTS* or other monoterpene synthases is one of the main rate-limiting steps. In a previous study, which currently stands as the sole investigation into the engineering of microorganisms for the *de novo* synthesis of α-terpineol, the researchers employed the upregulation of the monoterpene synthase pathway. This was achieved through the overexpression of *tHMG1*, *IDI1*, *ERG20* ^*ww*^, and *ERG9* genes in *Saccharomyces cerevisiae* with the aim of enhancing α-terpineol production (Zhang et al., 2019). The highest terpineol production reached 1.88 mg/L using a 5-L bioreactor in batch and fed-batch fermentation processes. To further improve the product titer, the expression level of *aTS* needs to be promoted by the increase in gene copy number and the utilization of other strong promoters (like P_*LDP1*_ and P_*GPD*_). Then, the expression of *ERG9*/*LPP1*/*DPP1* gene (catalyzing FPP to squalene and farnesol) could be downregulated to generate the GPP pool through the CRISPR/dCas9 system.

Unlike those for sesquiterpenes and other terpenoids, fewer synthetic biology efforts have been devoted to monoterpenes, likely due to their unstable property. Nonetheless, Zhuang et al. (2019) expressed 1,8-cineole synthase Hyp3 from *Hypoxylon* sp. E74060B and produced the highest titer of 14 mg/L in *R. toruloides*. In our previous study, we expressed a limonene synthase Ls1 from *Citrus limon*, and the maximum (R)-limonene titer was 37 mg/L (Liu S. et al., 2021). It suggested an incomparable titer between monoterpene and sesquiterpene. Albeit these previous works and the current study failed to achieve the production of monoterpenes in high titers, *R. toruloides* has currently been well documented as a host for further exploration of its capacity. In addition, various expression cassettes have been constructed during this study, which can be directly used in other pathway designs for the synthesis of various terpenes.

It is noteworthy that while analyzing the culturing medium for the purpose of α-terpineol production, it was observed that the diploid *R. toruloides* CGMCC 2.1389 exhibited a lower yield of α-terpineol compared to the haploid NP11 strain (Figure 2A). In our previous study, we also found that CGMCC 2.1389 would achieve more cell mass and lipid content under the same culturing condition (Zhang et al., 2022). These results indicate that the diploid CGMCC 2.1389 was more robust than the haploid NP11 in cell growth and lipid synthesis, leading to fewer carbon sources in the MVA pathway. It needs to be further verified through other experiments.

To verify the speculation that the ATMT mechanism of forming high copies would be interfered by the CRISPR/Cas9 editing system, more genes like *tHMG1*, *ERG9*, or *ERG20* could be integrated into the *R. toruloides* genome using different expression cassettes (plasmids CRT1.2(H) and HE-CRT1.2 as backbones) or transforming a same cassette by various methods (ATMT, LiAc, and electroporation). Subsequently, these randomly selected colonies will be cultured, and the copy number will be detected.

ATMT facilitates the insertion of multiple T-DNAs into the same chromosomal site (Tzfira, 2004), which causes the random integration of more than one copy into the *R. toruloides* genome. This mechanism confuses the integration loci yet is useful in producing more effectively designed strains (Figures 4, 6). Meanwhile, *R. toruloides* is a nonconventional yeast belonging to basidiomycetes. This yeast has a well-developed non-homologous end-joining (NHEJ), which limits homologous recombination (HR). Ploessl et al. (2022) discovered that the NHEJ disruption in a NHEJ-proficient host could impede microbial factory performance. They introduced a CRISPR platform that lowered the indel nuclease system enabling accurate repair to improve the HR efficiency without NHEJ disruption. This strategy might be an effective measure in *R. toruloides* homology-directed repair.

In conclusion, this is the first study to synthesize α-terpineol in *R. toruloides* with the best yield of 1.5 mg/L. Modular engineering of *R. toruloides* was introduced, which proved to be beneficial and necessary in monoterpenoid production. This study also indicated that the ATMT mechanism of forming high copies would be interfered by the CRISPR/Cas9 editing system, providing a valuable reference for other gene regulation through the same editing method.

## Data Availability

The datasets presented in this study can be found in online repositories. The names of the repository/repositories and accession number(s) can be found in the article/Supplementary Material.

## References

[B1] Alonso-Gutierrez J. Chan R. Batth T. S. Adams P. D. Keasling J. D. Petzold C. J. (2013). Metabolic engineering of *Escherichia coli* for limonene and perillyl alcohol production. Metab. Eng. 19, 33–41. 10.1016/j.ymben.2013.05.004 23727191

[B2] Christianson D. W. (2007). Roots of biosynthetic diversity. Science 316 (5821), 60–61. 10.1126/science.1141630 17412944

[B3] Daletos G. Katsimpouras C. Stephanopoulos G. (2020). Novel strategies and platforms for industrial isoprenoid engineering. Trends Biotechnol. 38 (7), 811–822. 10.1016/j.tibtech.2020.03.009 32359971

[B4] Geiselman G. M. Zhuang X. Kirby J. Tran-Gyamfi M. B. Prahl J.-P. Sundstrom E. R. (2020). Production of ent-kaurene from lignocellulosic hydrolysate in *Rhodosporidium toruloides* . Microb. Cell Fact. 19, 24. 10.1186/s12934-020-1293-8 32024522 PMC7003354

[B5] Ignea C. Pontini M. Maffei M. E. Makris A. M. Kampranis S. C. (2014). Engineering monoterpene production in yeast using a synthetic dominant negative geranyl diphosphate synthase. ACS Synth. Biol. 3 (5), 298–306. 10.1021/sb400115e 24847684

[B6] Jiao X. Lyu L. Zhang Y. Huang Q. Zhou R. Wang S. (2021). Reduction of lipid-accumulation of oleaginous yeast *Rhodosporidium toruloides* through CRISPR/Cas9-mediated inactivation of lipid droplet structural proteins. FEMS Microbiol. Lett. 368 (16), fnab111. 10.1093/femsle/fnab111 34410383

[B7] Jiao X. Zhang Y. Liu X. Zhang Q. Zhang S. Zhao Z. K. (2019). Developing a CRISPR/Cas9 system for genome editing in the basidiomycetous yeast *Rhodosporidium toruloides* . Biotechnol. J. 14 (7), 1900036. 10.1002/biot.201900036 31066204

[B8] Khaleel C. Tabanca N. Buchbauer G. (2018). α-Terpineol, a natural monoterpene: a review of its biological properties. Open Chem. 16, 349–361. 10.1515/chem-2018-0040

[B9] Kirby J. Geiselman G. M. Yaegashi J. Kim J. Zhuang X. Tran-Gyamfi M. B. (2021). Further engineering of *R. toruloides* for the production of terpenes from lignocellulosic biomass. Biotechnol. Biofuels 14, 101. 10.1186/s13068-021-01950-w 33883010 PMC8058980

[B10] Li Y. Zhao Z. K. Bai F. (2007). High-density cultivation of oleaginous yeast *Rhodosporidium toruloides* Y4 in fed-batch culture. Enzyme Microb Tech 41 (3), 312–317. 10.1016/j.enzmictec.2007.02.008

[B11] Lin X. Wang Y. Zhang S. Zhu Z. Zhou Y. J. Yang F. (2014). Functional integration of multiple genes into the genome of the oleaginous yeast *Rhodosporidium toruloides* . FEMS Yeast Res. 14 (4), 547–555. 10.1111/1567-1364.12140 24495153

[B12] Lin X. Yang F. Zhou Y. Zhu Z. Jin G. Zhang S. (2012). Highly-efficient colony PCR method for red yeasts and its application to identify mutations within two leucine auxotroph mutants: colony PCR for red yeasts. Yeast 29 (11), 467–474. 10.1002/yea.2926 23065821

[B13] Liu S. Zhang M. Ren Y. Jin G. Tao Y. Lyu L. (2021a). Engineering *Rhodosporidium toruloides* for limonene production. Biotechnol. Biofuels 14, 243. 10.1186/s13068-021-02094-7 34937561 PMC8697501

[B14] Liu Y. Ma X. Liang H. Stephanopoulos G. Zhou K. (2021b). Monoterpenoid biosynthesis by engineered microbes. J Ind Microbiol Biot 48 (9), kuab065. 10.1093/jimb/kuab065 PMC8788732 34601590

[B15] Liu Y. G. Chen Y. (2007). High-efficiency thermal asymmetric interlaced PCR for amplification of unknown flanking sequences. BioTechniques 43 (5), 649–656. 10.2144/000112601 18072594

[B16] Ploessl D. Zhao Y. Cao M. Ghosh S. Lopez C. Sayadi M. (2022). A repackaged CRISPR platform increases homology-directed repair for yeast engineering. Nat. Chem. Biol. 18, 38–46. 10.1038/s41589-021-00893-5 34711982

[B17] Ratledge C. Wynn J. P. (2002). The biochemistry and molecular biology of lipid accumulation in oleaginous microorganisms. Adv. Appl. Microbiol. 51, 1–51. 10.1016/s0065-2164(02)51000-5 12236054

[B18] Ren Y. Liu S. Jin G. Yang X. Zhou Y. J. (2020). Microbial production of limonene and its derivatives: achievements and perspectives. Biotechnol. Adv. 44, 107628. 10.1016/j.biotechadv.2020.107628 32882371

[B19] Sales A. Felipe L. de O. Bicas J. L. (2020). Production, properties, and applications of α-terpineol. Food Bioprocess Technol. 13 (8), 1261–1279. 10.1007/s11947-020-02461-6

[B20] Sales A. Paulino B. N. Pastore G. M. Bicas J. L. (2018). Biogeneration of aroma compounds. Curr. Opin. Food Sci. 19, 77–84. 10.1016/j.cofs.2018.03.005

[B21] Smythe E. Ayscough K. R. (2003). The Ark1/Prk1 family of protein kinases: regulators of endocytosis and the actin cytoskeleton. EMBO Rep. 4 (3), 246–251. 10.1038/sj.embor.embor776 12634840 PMC1315904

[B22] Tzfira T. (2004). *Agrobacterium* T-DNA integration: molecules and models. Trends Genet. 20 (8), 375–383. 10.1016/j.tig.2004.06.004 15262410

[B23] Unger T. Jacobovitch Y. Dantes A. Bernheim R. Peleg Y. (2010). Applications of the Restriction Free (RF) cloning procedure for molecular manipulations and protein expression. J. Struct. Bio 172 (1), 34–44. 10.1016/j.jsb.2010.06.016 20600952

[B24] Vespermann K. A. C. Paulino B. N. Barcelos M. C. S. Pessôa M. G. Pastore G. M. Molina G. (2017). Biotransformation of α- and β-pinene into flavor compounds. Appl. Microbiol. Biotechnol. 101 (5), 1805–1817. 10.1007/s00253-016-8066-7 28105487

[B25] Walls L. E. Otoupal P. Ledesma-Amaro R. Velasquez-Orta S. B. Gladden J. M. Rios-Solis L. (2023). Bioconversion of cellulose into bisabolene using *Ruminococcus flavefaciens* and *Rhodosporidium toruloides* . Bioresour. Technol. 368, 128216. 10.1016/j.biortech.2022.128216 36347482

[B26] Wen Z. Zhang S. Odoh C. K. Jin M. Zhao Z. K. (2020). *Rhodosporidium toruloides* - a potential red yeast chassis for lipids and beyond. FEMS Yeast Res. 20 (5), foaa038. 10.1093/femsyr/foaa038 32614407 PMC7334043

[B27] Zhang C. Li M. Zhao G.-R. Lu W. (2019). Alpha-Terpineol production from an engineered *Saccharomyces cerevisiae* cell factory. Microb. Cell Fact. 18, 160. 10.1186/s12934-019-1211-0 31547812 PMC6757357

[B28] Zhang Y. Kamal R. Li Q. Yu X. Wang Q. Zhao Z. K. (2022). Comparative fatty acid compositional profiles of *Rhodotorula toruloides* haploid and diploid strains under various storage conditions. Fermentation 8 (9), 467. 10.3390/fermentation8090467

[B29] Zhao Y. Song B. Li J. Zhang J. (2022). *Rhodotorula toruloides*: an ideal microbial cell factory to produce oleochemicals, carotenoids, and other products. World J. Microbiol. Biotechnol. 38, 13. 10.1007/s11274-021-03201-4 34873661

[B30] Zhou Y. J. Gao W. Rong Q. Jin G. Chu H. Liu W. (2012). Modular pathway engineering of diterpenoid synthases and the mevalonic acid pathway for miltiradiene production. J. Am. Chem. Soc. 134 (6), 3234–3241. 10.1021/ja2114486 22280121

[B31] Zhuang X. Kilian O. Monroe E. Ito M. Tran-Gymfi M. B. Liu F. (2019). Monoterpene production by the carotenogenic yeast *Rhodosporidium toruloides* . Microb. Cell Fact. 18, 54. 10.1186/s12934-019-1099-8 30885220 PMC6421710

