# Supplementary material for: Modularly engineering Rhodotorula toruloides for α-terpineol production
Source: Front Bioeng Biotechnol. 2024 Jan 19;11:1310069. doi: 10.3389/fbioe.2023.1310069 (PMC10835275; doi:10.3389/fbioe.2023.1310069)
Supplement: Supplementary file 1 [file Table1.PDF]

# Supplementary Tables

**Table S1** Strains used in this study.

| Strains | Description | Resource |
|---|---|---|
| *E. coli* DH10B | *$F^-$, mcaA, rpsL, nupG, Φ80dlacZΔM15, ΔlacX74, recA1, endA1, araD139, Δ (ara, leu)7697, galE15, galK, $\lambda^-$, Δ(mrr-hsdRMS-mcrBC)* | Takara |
| *R. toruloides* NP11 | *MAT A1* | [1] |
| *Agrobacterium tumefaciens* AGL1 | AGL0 *recA::bla, pTiBo542DT, $Mop^+$, CbR* | [2] |
| 11aTS | NP11 *aTS, NAT*<br>（NP11 carrying the aTS-NAT） | |
| 4aTS | CGMCC 2.1389 *aTS, NAT*<br>（CGMCC 2.1389 carrying the aTS-NAT） | |
| 5C | 11aTS-5 *SaCas9, crtΔ, BLE*<br>（11aTS-5 carrying the SaCas9-CRT1.2(B)） | |
| 5L | 11aTS-5 *SaCas9, ldp1Δ, BLE*<br>（11aTS-5 carrying the SaCas9-LDP1.2） | |
| 5HE | 11aTS-5 *tHMG1, $ERG20^{ww}$, HYG*<br>（11aTS-5 carrying the tHMG1-ERG20ww） | |
| 5C2HE | 5C2 *tHMG1, $ERG20^{ww}$, HYG*<br>（5C2 carrying the tHMG1-ERG20ww） | This study |
| 5L6C | 5L6 *crtΔ, HYG*<br>（5L6 carrying the CRT1.2(H)） | |
| 5L6HE | 5L6 *tHMG1, $ERG20^{ww}$, HYG*<br>（5L6 carrying the tHMG1-ERG20ww） | |
| 5L6CHE | 5L6 *tHMG1, $ERG20^{ww}$, crtΔ, HYG*<br>（5L6 carrying the HE-CRT1.2） | |
| 5L6CHED | 5L6 *tHMG1, $ERG20^{ww}$, crtΔ, HYG*<br>（5L6CHE carrying the HE-CRT1.2） | |

**Table S2** Plasmids used in this study.

| Plasmids | Description | Resource |
|---|---|---|
| pJX15 | pZPK-$P_{PGK}$-*HYG*-P2A-*BLE*-P2A-*NAT*-$T_{HSP}$ | [3] |
| pZPK-Cas9 | pZPK-$P_{PGK}$-*BLE*-$T_{NOS}$-$P_{GPD}$-*CAS9*-$T_{HSP}$ | [4] |
| pZPK-Car2.X-sgRNA | pZPK-$P_{GPD}$-*NAT*-Tnos-U6b-CRT1.2-sgRNA | [4] |
| p424-tHMG1 | / | [5] |
| PGK-HYG-THSP | pZPK-$P_{PGK}$-*HYG*-$T_{HSP}$ | [2] |
| aTS-NAT | pZPK-$P_{PGK}$-*aTS*-P2A-*NAT*-$T_{HSP}$ | |
| SaCas9-CRT1.2(B) | pZPK-$P_{PGK}$-*BLE*-$T_{NOS}$-$P_{GPD}$-*SaCas9*-$T_{HSP}$-5stRNA-CRT1.2-sgRNA | |
| SaCas9-LDP1.2 | pZPK-$P_{PGK}$-*BLE*-$T_{NOS}$-$P_{GPD}$-*SaCas9*-$T_{HSP}$-5stRNA-LDP1.2-sgRNA | This study |
| HE-CRT1.2 | pZPK-$P_{PGK}$-*tHMG1*-F2A-*ERG20*$^{ww}$-P2A-*HYG*-$T_{HSP}$-5stRNA-CRT1.2-sgRNA | |
| CRT1.2(H) | pZPK-$P_{PGK}$-*HYG*-$T_{NOS}$-5stRNA-CRT1.2-sgRNA | |
| tHMG1-ERG20ww | pZPK-$P_{PGK}$-*tHMG1*-F2A-*ERG20*$^{ww}$-P2A-*HYG*-$T_{HSP}$ | |

**Table S3** Primers used in this study.

| Primers | Sequence (5'-3') | Description |
|---|---|---|
| PGK-aTS-F | ttcacagcaactcacccgtccaactcccaccctcccccgtgcagcccaccatggccctctcgatgctctcgtcgatcccg | Amplification of the $P_{PGK}$-*aTS*-P2A fragment |
| aTS-P2A-R | tcgacgtcgccagcctgcttgaggagcgagaagttggtagcgcccgagccctcgaacgagagcgacgggatcggctcgac | |
| PGK-tHMG1-F | ctcacccgtccaactcccaccctcccccgtgcagcccaccatggctgcagaccaattggtg | Amplification of the $P_{PGK}$-*tHMG1*-F2A fragment from the plasmid p424-tHMG1 |
| tHMG1-F2A-R | agggccagggttcgactcgacgtcgccagcgagcttgaggaggtcgaagttgagggtctgcttgacgcccgagccggatttaatgcaggtgacgg | |
| F2A-ERG20-F | ggctcgggcgtcaagcagaccctcaacttcgacctcctcaagctcgctggcgacgtcgagtcgaaccctggccctatggcttcagaaaaagaaat | Amplification of the F2A-*ERG20*-P2A fragment from *S. cerevisiae* genome |
| ERG20-P2A-R | cagcctgcttgaggagcgagaagttggtagcgcccgagcctttgcttctcttgtaaactttg | |
| HYG-THSP-R | ctacacaggtcggatgcgaggtgagacggggcggaatcgtctagtgatggtgatggtggtg | Amplification of the $P_{PGK}$-*tHMG1*-F2A-*ERG20*$^{ww}$-P2A-*HYG*-$T_{HSP}$ fragment |
| ERG20Fm(96w) | caggcttactggttggtcgccgatgatatg | Double site mutation of *ERG20* |
| ERG20Nm(127w) | ctaacatgaatgcgtcccagatggcaatttc | |
| 5S-tRNA-F1 | gcagcatgcaagcttggagcttgagcttggatctgcggccataccgcgatgaacacaccgcgtctcgtccgatccgcgaagttaagcatcgcaggggccagaga | Replace the U6b promotor of pZPK-Car2.X-sgRNA plasmid to 5s-tRNA promotor |
| 5S-tRNA-R1 | aacaggtgccgtgccacttggccacaccgccacctgcggcagcacagtgttctcgcctggtcacccacggcaatactctctggcccctgcgatgcttaacttcg | |
| 5s-F | ccgatctgcggccataccgcg | Amplification the fragment of 5stRNA-CRT1.2/LDP1.2-sgRNA |
| 5s-LDP1.2-sg-R | gttttagtagattctgtttccagagtactaaaacgggccgtctggaacgcggcatcgacggtgaaggggatcgaac | |
| 5s-CRT1.2-sg-R | gttttagtagattctgtttccagagtactaaaacactgggcgaggcgagcggcgcgacggtgaaggggatcgaac | |
| THSP-5s-F | cggttcacatagtcaaagatgcagtgcagagaagtgcgcgccgatctgcggccataccgc | Amplification the fragment of $T_{HSP}$-5stRNA-sgRNA |
| sgRNA-R | ctgatagtttaaactgaaggcgggaaacgacaatctgatagacataaaaaacaaaaaaatc | |
| ANF1 | ctggttgtatttcgcgggc | Amplification of *ACTIN* gDNA |
| ANR1 | gagcagctctttaccaatgc | |
| Actin-F1 | cgagtcagtggctgcgtcag | Amplification of *ACTIN* for qPCR |
| Actin-R1 | ctgacggttccgttcgctc | |
| LB-F | ggcaggatatattgtggtg | Amplification of $P_{PGK}$-*HYG*-$T_{HSP}$ |
| RB-R | gtttacccgccaatatatcc | |
| HYG-WF1 | atgccggagctcacggcgac | Amplification of *HYG* for qPCR |
| HYG-WR1 | gtgtcatccatgaccgtttg | |
| aTS-0a | cttgtcgaggcggcgggtgtaggtc | Genome Walking |
| aTS-1a | cgatggactgctgagtggcacctggtcccagatggtggggtggtag | |
| aTS-2a | gccttgcaggaggaggacttgatgatg | |
| LAD-1 | acgatggactccagagcggccgcvnvnnnggaa | |
| LAD-2 | acgatggactccagagcggccgcbnbnnnggtt | |
| LAD-3 | acgatggactccagagcggccgcvvnvnnnccaa | |
| LAD-4 | acgatggactccagagcggccgcbdnbnnncggt | |
| AC1 | acgatggactccagag | |

**Table S4** Copy number calculation of gene *tHMG1* and $ERG20^{ww}$ in strains 5L6HE and 5L6CHE

| Strains | Copy No. | Genotype |
|---|---|---|
| 5L6HE1 | 2.1±0.49 | |
| 5L6HE2 | 1.7±0.20 | pZPK-$P_{PGK}$-*aTS*-P2A-*NAT*-$T_{HSP}$ |
| 5L6HE4 | 1.5±0.10 | pZPK-$P_{PGK}$-*BLE*-$T_{NOS}$-$P_{LDP1}$-*SaCas9*-$T_{HSP}$-5stRNA-LDP1.2-sgRNA |
| 5L6HE5 | 1.6±0.08 | pZPK-$P_{PGK}$-*tHMG1*-F2A-$ERG20^{ww}$-P2A-*HYG*-$T_{HSP}$ |
| 5L6HE6 | 1.5±0.20 | |
| 5L6CHE1 | 0.7±0.08 | |
| 5L6CHE2 | 1.0±0.27 | pZPK-$P_{PGK}$-*aTS*-P2A-*NAT*-$T_{HSP}$ |
| 5L6CHE4 | 0.8±0.04 | pZPK-$P_{PGK}$-*BLE*-$T_{NOS}$-$P_{LDP1}$-*SaCas9*-$T_{HSP}$-5stRNA-LDP1.2-sgRNA |
| 5L6CHE5 | 0.8±0.05 | pZPK-$P_{PGK}$-*tHMG1*-F2A-$ERG20^{ww}$-P2A-*HYG*-$T_{HSP}$-5stRNA-CRT1.2-sgRNA |
| 5L6CHE6 | 0.7±0.09 | |

## Reference


(1) Zhu Z, Zhang S, Liu H, Shen H, Lin X, Yang F, Zhou YJ, Jin G, Ye M, Zou H, Zhao ZK (2012) A multi-omic map of the lipid-producing yeast *Rhodosporidium toruloides*. Nat Commun 3:1112. https://doi.org/10.1038/ncomms2112.

(2) Lin X, Wang Y, Zhang S, Zhu Z, Zhou YJ, Yang F, Sun W, Wang X, Zhao ZK (2014) Functional integration of multiple genes into the genome of the oleaginous yeast *Rhodosporidium toruloides*. FEMS Yeast Res 14(4):547–555. https://doi.org/10.1111/1567-1364.12140

(3) Jiao X, Zhang Q, Zhang S, Yang X, Wang Q, Zhao ZK (2018) Efficient co-expression of multiple enzymes from a single promoter mediated by virus 2A sequence in the oleaginous yeas*t Rhodosporidium toruloide*s. FEMS Yeast Res 18(8):foy086. https://doi.org/10.1093/femsyr/foy086.

(4) Jiao X, Zhang Y, Liu X, Zhang Q, Zhang S, Zhao ZK (2019) Developing a CRISPR/Cas9 system for genome editing in the basidiomycetous yeast *Rhodosporidium toruloides*. Biotechnol J 14(7):1900036. https://doi.org/10.1002/biot.201900036

(5) Zhou YJ, Gao W, Rong Q, Jin G, Chu H, Liu W, Yang W, Zhu Z, Li G, Zhu G, Huang L, Zhao ZK (2012) Modular pathway engineering of diterpenoid synthases and the mevalonic acid pathway for miltiradiene production. J Am Chem Soc 134(6):3234–3241. https://doi.org/10.1021/ja2114486
