# Supplementary material for: Modularly engineering Rhodotorula toruloides for α-terpineol production
Source: Front Bioeng Biotechnol. 2024 Jan 19;11:1310069. doi: 10.3389/fbioe.2023.1310069 (PMC10835275; doi:10.3389/fbioe.2023.1310069)
Supplement: Supplementary file 2 [file Image1.PDF]

Supplementary Figures

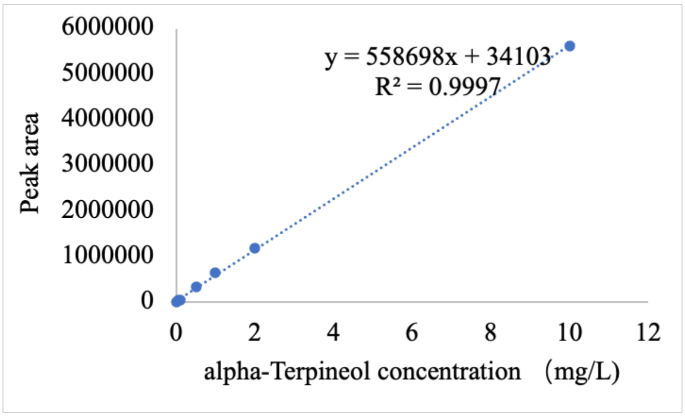

Figure S1 Standard curve of alpha-terpineol quantification taken out by GC-MS.

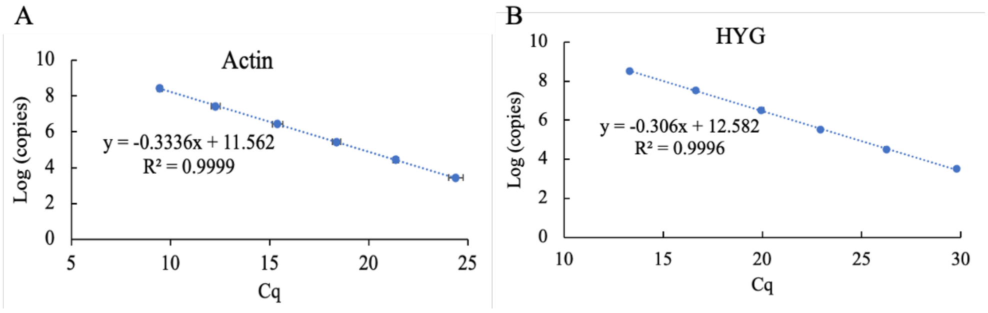

Figure S2 Standard curves generated by PCR products. A, *ACTIN* gene sequence (about 3000 bp) amplified from NP11 genomic DNA; B, *HYG* gene sequence (about 3000 bp) amplified from plasmid PGK-HYG-THSP. Cq, cycle threshold. The absolute copy number under each concentration of PCR products was calculated and taked the logarithm. Experiments were performed in triplicate.

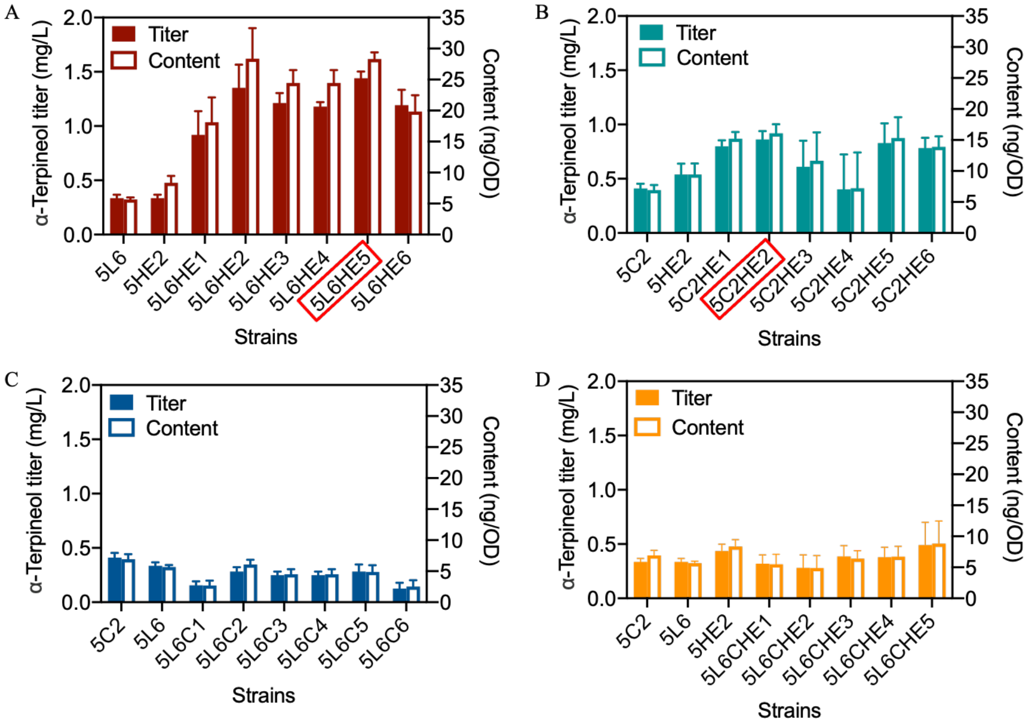

Figure S3 Production of alpha-terpineol using combined strategies. Experiments were performed in triplicate.
